# Mechanisms of Keap1/Nrf2 modulation in bacterial infections: implications in persistence and clearance

**DOI:** 10.3389/fimmu.2024.1508787

**Published:** 2024-12-23

**Authors:** Marco Antonio Romero-Durán, Octavio Silva-García, Jose Manuel Perez-Aguilar, Víctor Manuel Baizabal-Aguirre

**Affiliations:** ^1^ Centro Multidisciplinario de Estudios en Biotecnología, Facultad de Medicina Veterinaria y Zootecnia, Universidad Michoacana de San Nicolás de Hidalgo, Morelia, Mexico; ^2^ School of Chemical Sciences, Meritorious Autonomous University of Puebla (BUAP), University City, Puebla, Mexico

**Keywords:** KEAP1, Nrf2, bacteria, infection, inflammatory mechanisms, oxidative stress

## Abstract

Pathogenic bacteria trigger complex molecular interactions in hosts that are characterized mainly by an increase in reactive oxygen species (ROS) as well as an inflammation-associated response. To counteract oxidative damage, cells respond through protective mechanisms to promote resistance and avoid tissue damage and infection; among these cellular mechanisms the activation or inhibition of the nuclear factor E2-related factor 2 (Nrf2) is frequently observed. The transcription factor Nrf2 is considered the *master* regulator of several hundred cytoprotective and antioxidant genes. Under normal conditions, the Keap1/Nrf2 signaling protects the cellular environment by sensing deleterious oxygen radicals and inducing the expression of genes coding for proteins intended to neutralize the harmful effects of ROS. However, bacteria have developed strategies to harness Nrf2 activity to their own benefit, complicating the host response. This review is aimed to present the most recent information and probable mechanisms employed by a variety of bacteria to modulate the Keap1/Nrf2 activity in order to survive in the infected tissue. Particularly, those utilized by the Gram-positive bacteria *Staphylococcus aureus*, *Streptococcus pneumoniae*, *Listeria monocytogenes*, and *Mycobacterium tuberculosis* as well as by the Gram-negative bacteria *Escherichia coli*, *Helicobacter pylori*, *Legionella pneumophila*, *Pseudomonas aeruginosa* and *Salmonella typhimurium*. We also discuss and highlight the beneficial impact of the Keap1/Nrf2 antioxidant and anti-inflammatory role in bacterial clearance.

## Introduction

The innate immune response is the first line of defense against infectious agents such as parasites, viruses, fungi, and bacteria. It involves a complex network of molecular interactions and cellular defense mechanisms between host and pathogen, intended not only to eliminate the pathogen (resistance) but also to limit tissue damage and infection (tolerance) without directly targeting the pathogen. Under normal conditions, these complex mechanisms give rise to a successful resolution of the infection and a recovery of the tissue homeostasis (1). Among the different mechanisms currently studied and known to contribute to immune tolerance associated with infections caused by bacterial pathogens, the activation of nuclear transcription factor erythroid 2-related factor 2 (Nrf2) has been considered of central importance (2, 3). Nrf2 is regarded as the antioxidant and anti-inflammatory *master* regulator that protects cells from tissue damage caused by oxidative stress via an antioxidant defense response (4). Although the importance of Nrf2 as one of the multiple responses to infection is accepted worldwide, many questions remain unanswered, especially those related to oxidative stress and the molecular mechanisms involved in such regulation. In particular, one of these questions concerns how pathogenic Gram-positive and Gram-negative bacteria manipulate the Keap1/Nrf2 signaling pathway and how cells respond to limit bacterial survival and replication. Understanding these molecular mechanisms should be fundamental to design new therapeutic strategies to regulate and control infections, reducing tissue damage and its inflammatory consequences.

The main goal of this review is to present new findings that strongly support the regulatory role of the transcription factor Nrf2 during bacterial infections. The first section includes an overview of the Keap1/Nrf2 relationship to immune modulation and inflammation, and a detailed description of the signaling pathway mechanism. The second and third parts are devoted to concisely describe the molecular mechanisms employed by the Gram-positive bacteria *Staphylococcus aureus*, *Streptococcus pneumoniae*, *Listeria monocytogenes*, and *Mycobacterium tuberculosis* and by the Gram-negative bacteria *Escherichia coli*, *Helicobacter pylori*, *Legionella pneumophila*, *Pseudomonas aeruginosa*, and *Salmonella typhimurium* in the activation or inhibition of the Keap1/Nrf2 signaling.

## The role of Keap1/Nrf2 in the inflammatory response

Nrf2 activation is particularly relevant in inflammation, which is the most essential process within the innate immune response. Inflammation started with the recognition of pathogen-associated molecular patterns (PAMPs) or cell damage-associated molecular patterns (DAMPs) by pattern recognition receptors (PRRs) such as transmembrane Toll-like receptors (TLRs) and intracellular nucleotide oligomerization domain-like receptors (NODs or NLRs). These PRRs promote the expression of inflammatory mediators such as chemokines, cytokines, eicosanoids, proteases, and reactive oxygen species (ROS) (5). The most relevant ROS include the superoxide anion, hydrogen peroxide, and hydroxyl radicals formed because of the partial reduction of oxygen (6). Although low levels of ROS are essential to eliminate pathogens, an excess can induce the generation of oxidative stress as a result of an imbalance between oxidizing and antioxidant molecules. Since ROS are very unstable and may quickly react and damage biological macromolecules including DNA, proteins, and lipids, cells must maintain tight control over ROS to keep cellular homeostasis. Structure alteration of these biomolecules caused by ROS may lead to the development of chronic and degenerative diseases, including Alzheimer’s, Parkinson’s, rheumatoid arthritis, premature aging, cancer, cardiovascular and pulmonary disorders, as well as viral, parasitic and bacterial infections (7–10).

During an infection process, cells of the immune system, including lymphocytes, dendritic cells, macrophages, and neutrophils sense pathogenic microorganisms that are subsequently engulfed and eliminated by the formation of phagosomal structures. Once phagosomes mature by fusion with endosomes and lysosomes, a ROS oxidative burst develops (11). A sustained increase in ROS may be detrimental and even lead to cell death by apoptosis. In addition, high concentrations of ROS activate signaling pathways involved in the production of free radicals and inflammation by activation of the transcription factor nuclear factor kappa B (NF-κB) that induces the expression of the proinflammatory cytokines tumor necrosis factor-alpha (TNF-α), interleukin-6 (IL-6), IL-1β, and many others (12, 13). In this context, activation of antioxidant and anti-inflammatory genes is of vital importance to counteract the inflammation and maintain redox homeostasis. Among the large battery of biomolecules that contribute to restrain the deleterious effects of ROS are worth to mention reduced glutathione (GSH), superoxide dismutase (SOD), catalase (CAT) and phase II detoxifying enzymes such as heme oxygenase-1 (HO-1), NADPH: Quinone Oxidoreductase 1 (NQO-1), Glutathione S-Transferase (GST), thioredoxin and peroxiredoxin systems, NADPH generation systems, and iron metabolism (14). Notably, Nrf2 regulates the expression of genes encoding most of these antioxidant and detoxifying enzymes.

## The Nrf2 signaling pathway

**Figure 1:**
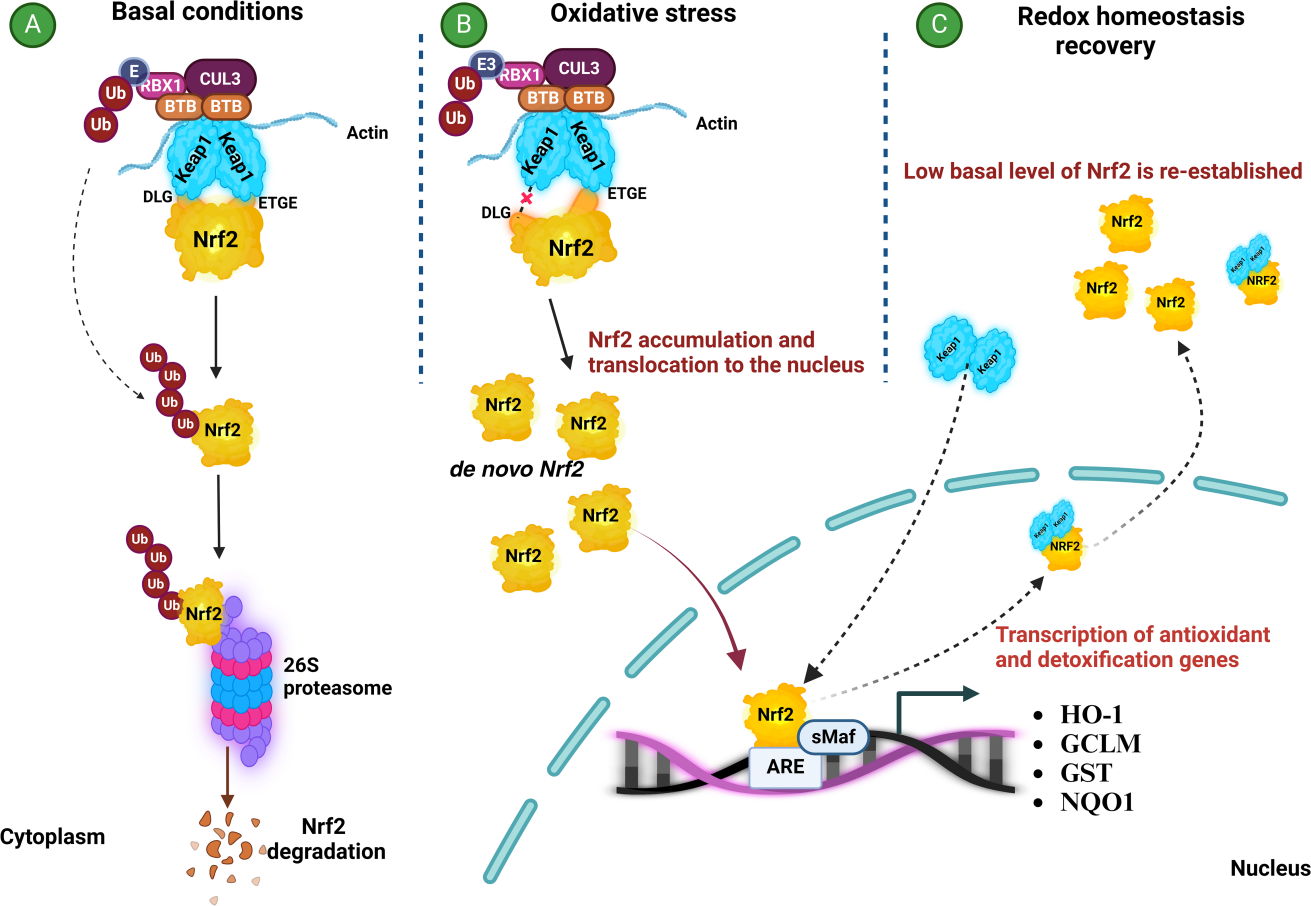
The NRF2/KEAP1 signaling pathway adapted from [17]. **(A)** Under basal conditions, Nrf2 remains inactive in the cytoplasm bound to its regulatory protein Keap1. This protein is associated with the CUL3-RBX1 complex, allowing the transfer of ubiquitin (Ub) to the Lys residues located between the DLG and ETGE motifs of the Nrf2 Neh2 domain. Then, degradation of Nrf2 occurs by ubiquitination and recognition by the proteasome 26S. **(B)** During oxidative stress, Cys residues at KEAP1 are oxidized at their BTB and/or IVR domains, inducing dissociation between Nrf2 and Keap1. Nrf2 is released to the cytoplasm and, after accumulation, it is translocated to the nucleus where it binds to the small aponeurotic muscle fibrosarcoma (sMaf) proteins and induces the expression of antioxidant and detoxifying genes coding for enzymes that neutralize ROS and restore redox balance. **(C)** To restore redox balance, Keap1 is translocated to the nucleus and translocates Nrf2 back to the cytoplasm. This controls the time of the antioxidant and detoxifying response. ARE, antioxidant response element; BTB, Broad complex Tramtrack and Bric-a-Brac; Cul3, Cullin 3 protein; DLG, low-affinity motif of the Nrf2 Neh2 domain; E3, E3 ubiquitin ligase; ETGE, high-affinity motif of the Nrf2 Neh2 domain; GCLM, Glutamate-cysteine ligase modifier subunit; GST, Glutathione S-Transferase; HO-1, Heme Oxygenase-1; Keap1, Kelch-like ECH-associated protein 1; NQO-1, NADPH: Quinone Oxidoreductase 1; Nrf2, nuclear transcription factor erythroid 2-related factor 2; RBX1, RING box protein-1; sMaF, small aponeurotic muscle fibrosarcoma proteins; Ub, ubiquitin.

Nrf2 belongs to the cap ‘n’ collar (CNC) subfamily of transcription factors with an essential leucine zipper (bZip) located at its C-terminal region (15). Structurally, it comprises seven highly conserved domains called Neh (Nrf2-ECH homology). Under normal physiological conditions, Nrf2 activity is regulated by its cytoplasmic interactions with the two Kelch domains of the homodimeric Kelch-like ECH-associated protein Keap1. The homodimeric Keap1 interacts with the high-affinity (ETGE) and low-affinity (DLG) motifs of the Neh2 domain of Nrf2, which allows Keap1 to function as an adaptor of the RING box protein 1 (RBX1)-associated ubiquitin E2 ligase Cullin-3 (CUL3). The complex Keap1-RBX1-CUL3 ubiquitinates Nrf2 at Lys residues located between the DLG and ETGE motifs, priming its degradation by the proteasome 26S (**Figure 1A**) (16, 17). However, under oxidative or electrophilic conditions, the Cys thiol groups of Cys158 present in the Keap1 Broad complex Tramtrack and Bric-a-Brac (BTB) domain or that of Cys288 at the intervening region (IVR) are oxidized. This induces a mechanism of dissociation between Nrf2 and Keap1 that is different from the “Hinge-Latch” mechanism proposed by Horie et al. (2021) for protein-protein interaction inhibitors, thus facilitating the cytoplasmic accumulation of Nrf2 and its subsequent translocation to the nucleus (18). Once in the nucleus, Nrf2 binds to small aponeurotic muscle fibrosarcoma (sMaf) proteins and induces the expression of antioxidant response element (ARE)-driven genes encoding antioxidant and detoxifying enzymes that neutralize ROS, electrophiles, hydroperoxides, carbonyls, quinones, and exogen stimuli such as xenobiotics and UV light thereby restoring the redox balance (**Figure 1B**) (19). Recovery from oxidative stress is achieved when Keap1 translocates to the nucleus and promotes Nrf2 nuclear export. This mechanism restores normal physiological levels of Nrf2 and prevents the antioxidant and detoxifying response from being unnecessarily prolonged (**Figure 1C**).

Although Nrf2 is activated mainly by high ROS concentrations, several non-canonical mechanisms that regulate Nrf2 activity have also been described. One of them is characterized by phosphorylation of Nrf2 at Ser/Thr residues carried out by protein kinases such as ERK, p38, PKC, JNK, MSK1/2, and PI3K (20, 21). Another mechanism involves formation of a cytoplasmic macromolecular complex constituted by the enzyme glycogen synthase kinase-3β (GSK3β), Axin-1 and Cullin1-associated E3 ubiquitin ligase and beta-transducin repeats-containing proteins (β-TrCP), whose function is to remove low concentrations of ROS produced by normal cellular metabolism (22, 23). Interestingly, this mechanism also controls Nrf2 activity by promoting its degradation in a Keap1-independent manner, through a process that requires phosphorylation at Ser residues of the Neh6 domain (24). A third mechanism of Nrf2 activation involves the autophagy signaling pathway p62/Sequestosome 1 (SQSTM1; hereinafter referred to as p62) protein-dependent degradation of Keap1, in which many proteins are digested (4, 25).

Pharmacological activation of Nrf2 can be achieved through various compounds with electrophilic properties capable of covalently modifying cysteine (Cys) residues in Keap1. This chemical modification induces conformational changes that prevent the ubiquitination and subsequent proteasomal degradation of Nrf2 (26). Examples of such compounds include sulforaphane, chalcone, and dimethyl fumarate (DMF) (26–28). Alternatively, non-electrophilic compounds can activate this signaling pathway by reversibly inhibiting the Nrf2-Keap1 protein-protein interaction (PPI). These include compound 7, diaryl-triazole-derived compounds (27, 29), Nrf2 peptides containing ETGE or DLG motifs, p62-derived peptides, compounds derived from tetrahydroisoquinoline (THIQ), indoline, or related to 1,4-diaminonaphthalene or iminocoumarin-benzothiazole, among many others (27). Interestingly, the selective activation of Nrf2 by various chemical molecules has been shown to induce a cytoprotective effect against bacterial infections, as will be discussed in the following sections.

## Gram positive bacteria

### Staphylococcus aureus

**Figure 2:**
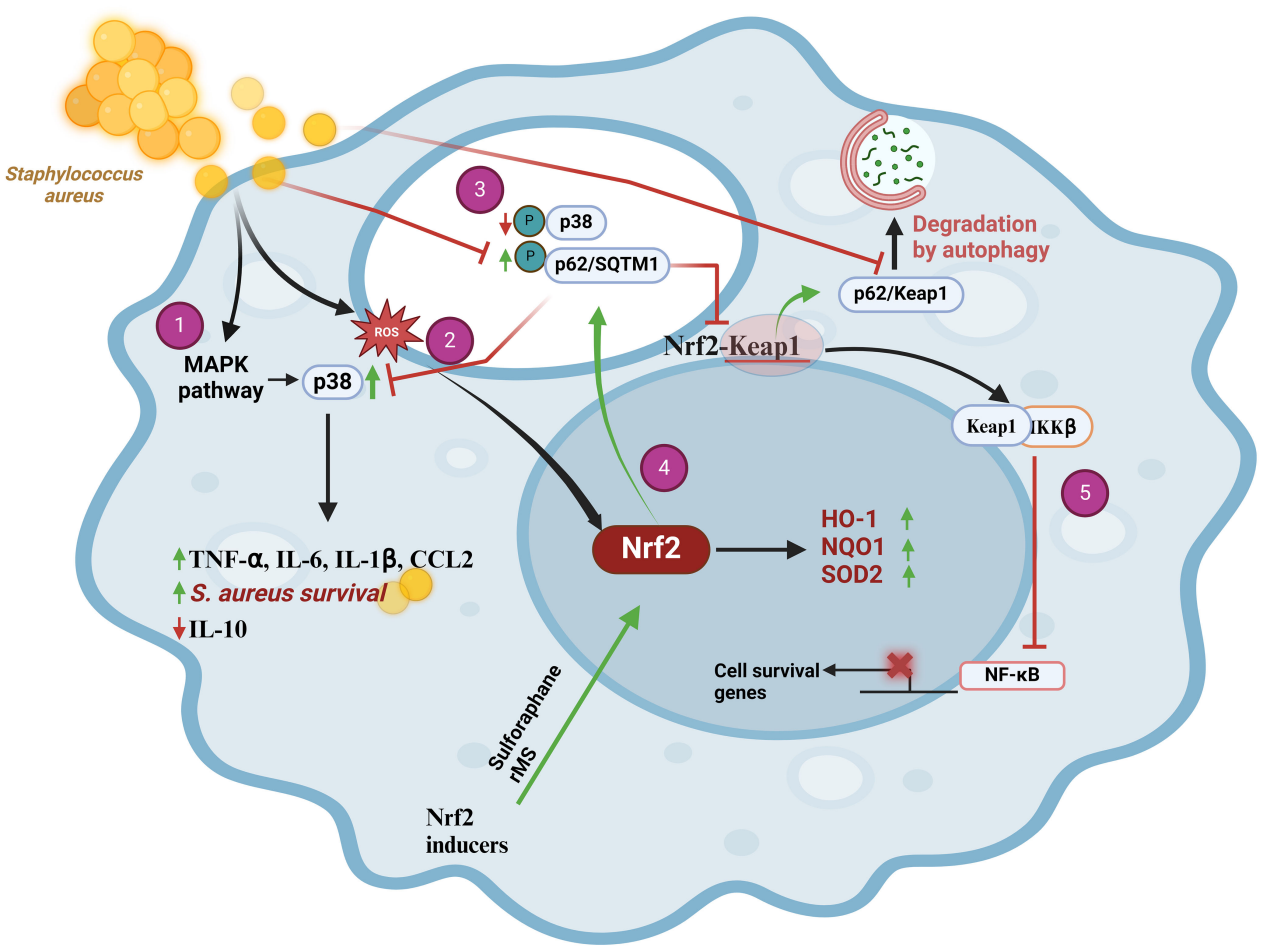
*Staphylococcus aureus* prevents Nrf2 pathway activation in THP-1 macrophages through upregulation of MAPK/p38 and downregulation of p62/SQTM1, scheme modified from [35]. (1) During the initial phase of infection, *S. aureus* causes an increase in ROS that activates Nrf2. (2) Upon internalization, *S. aureus* induces the expression of pro-inflammatory cytokines (TNF-α, IL-6, IL-1β, and CCL2) by activating the MAPK signaling pathway. This causes p38 phosphorylation that promotes bacterial cell survival and decreases the expression of the anti-inflammatory cytokine IL-10. (3) *S. aureus* manages to suppress the cellular antioxidant response by sequestering the p62/SQSTM1-Keap1/Nrf2 signaling pathways. This bacterium causes a decrease in p62/SQTM1 phosphorylation at Ser349, which diminishes its affinity for the Keap1 protein, thereby preventing formation of the p62-Keap1 complex and its degradation through the autophagy pathway. The final outcome of this mechanism is the repression of Nrf2. (4) Selective activation of Nrf2 decreased MAPK/p38 phosphorylation that increases p62 phosphorylation at Ser349 and its affinity for Keap1. Then, Keap1 is degraded via autophagy and Nrf2 translocates to nucleus to activate the antioxidant response, which could aid in *S. aureus* clearance. (5) Keap1 negatively regulates NF-κB by interacting with IKKβ. Ultimately, this stabilizes IκBα. CCL2, chemokine (C-C motif) ligand 2; HO-1, Heme Oxygenase-1; IKKβ, inhibitory kappa B kinase beta; IL-10, Interleukin 10; IL-6, interleukin-6; IL-1β, interleukin-1beta; Keap1, Kelch-like ECH-associated protein 1; MAPK, mitogen-activated protein kinase; NF-κB, nuclear factor kappa B; NQO-1 NADPH, Quinone Oxidoreductase 1; Nrf2, nuclear transcription factor erythroid 2-related factor 2; p38, p38 mitogen-activated protein kinases; p62/SQSTM1, p62 protein, also called sequestosome 1; ROS, reactive oxygen species; SOD, superoxide dismutase.

**Figure 3:**
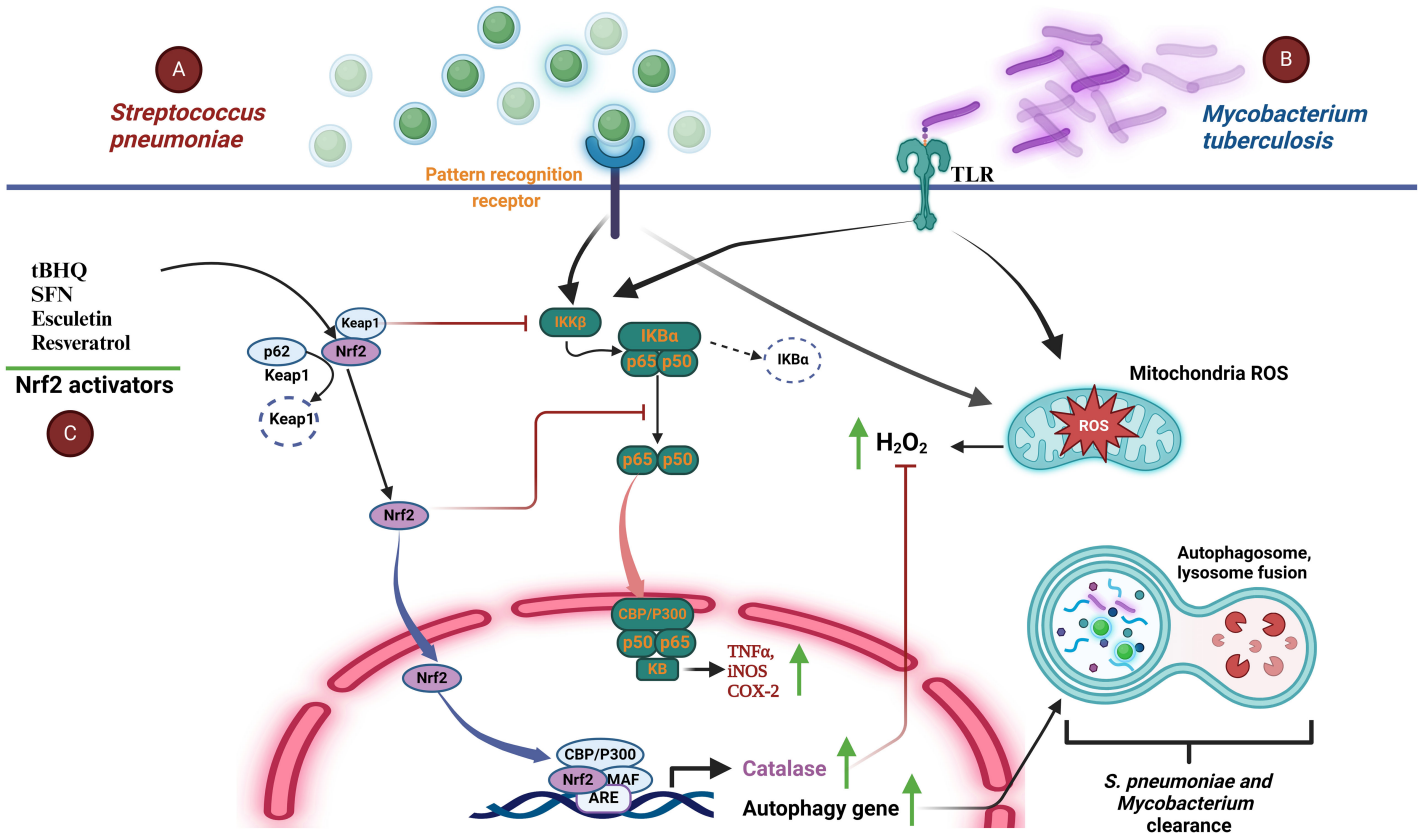
Activation of Nrf2 in *S. pneumoniae* and *M. tuberculosis* infections. **(A)** The inflammatory and redox response to *S. pneumoniae* infection (green spheres) in lung epithelial cells is initiated by their interaction with pattern recognition receptors (PRRs) that stimulate the activation of the inflammatory response mediated by NF-κB, which translocates to the nucleus and promotes the expression of TNF-α, iNOS, and COX-2. Simultaneously, *S. pneumoniae* induces the generation of mitochondrial reactive species (i.e. H_2_O_2_). An increment of H_2_O_2_ activates the transcription factor Nrf2. This in turn promotes expression of antioxidant and autophagy genes, allowing elimination of H_2_O_2_, promoting bacterial phagocytosis, and reducing the inflammatory response by blocking the nuclear translocation of NF-κB. **(B)** *M. tuberculosis* (purple rods) uses a mechanism very similar to that employed by *S. pneumoniae* because it interacts specifically with host cell TLRs, activating NF-κB-mediated inflammation. At the same time, the increase in H_2_O_2_ upon infection activates Nrf2, which promotes the expression of antioxidant enzymes thereby re-establishing cellular homeostasis, reducing inflammation, and favoring bacterial clearance through the autophagosome pathway. **(C)** Nrf2 activation by molecules such as SFN, TBHQ, and resveratrol, among others, restores the host redox imbalance, tissue damage and inflammation. These compounds are also able to regulate bacterial persistence and burden by inducing the expression of genes targeting autophagy. ARE, antioxidant response element; CBP, creb-binding protein; COX-2, Cyclooxygenase-2; H_2_O_2_, hydrogen peroxide; IκBα, inhibitor of nuclear factor kappa b; IKKβ, inhibitory kappa kinase beta; Keap1, kelch-like ECH-associated protein 1; NF-κB, factor nuclear factor kappa b; Nrf2, nuclear transcription factor erythroid 2-related factor 2; P300, e1a binding protein p300; PRRs, pattern recognition receptors; ROS, reactive oxygen species; SFN, sulforaphane; sMaF, small aponeurotic muscle fibrosarcoma proteins; TLRs, toll-like receptors; TNF-α, tumor necrosis factor-α; tBHQ, tert-butylhydroquinone; iNOS, inducible nitric oxide synthase.

**Figure 4:**
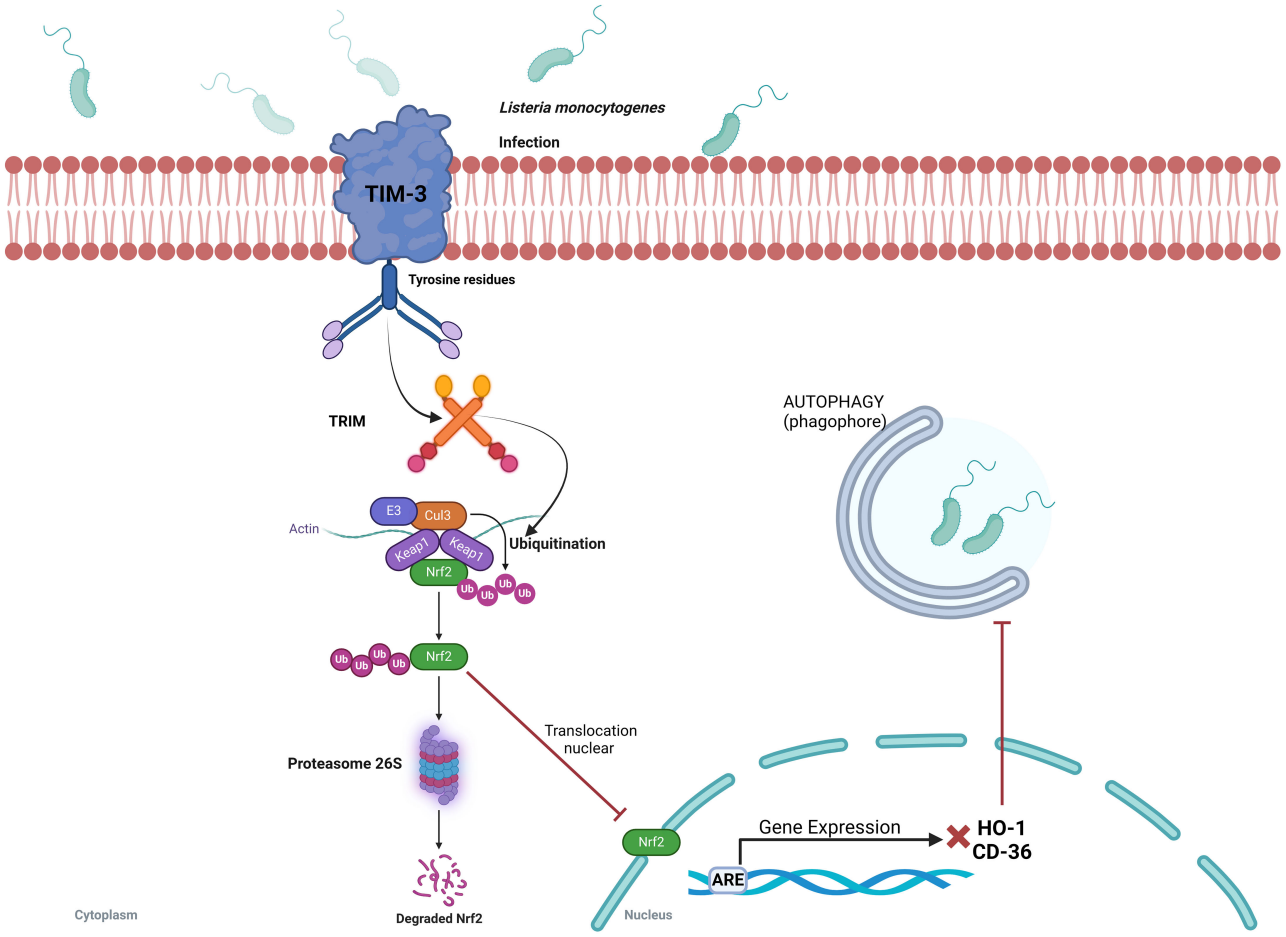
*Listeria monocytogenes* evades the host immune response by Tim-3-dependent inhibition of the Keap1/Nrf2 signaling pathway. Interaction of *L. monocytogenes* with Tim-3 (the macrophage type I membrane protein) induces ubiquitination and degradation of the downstream transcription factor Nrf2. A decrease in Nrf2 levels reduces the expression of the effector genes CD36 and HO-1, which decreases the phagocytic capacity of macrophages and favors bacterial survival. Restoration of Nrf2 levels reverses these effects, suggesting that Nrf2 activation may enhance bacterial clearance from infected tissues. ARE, antioxidant response element; CD-36, platelet glycoprotein 4; Cul3, Cullin 3 protein; E3, E3 ubiquitin ligase; HO-1, Heme oxygenase-1; Keap1, Kelch-like ECH-associated protein 1; Nrf2, nuclear transcription factor erythroid 2-related factor 2; TRIM, tripartite motif family proteins; Ub, ubiquitin.

**Figure 5:**
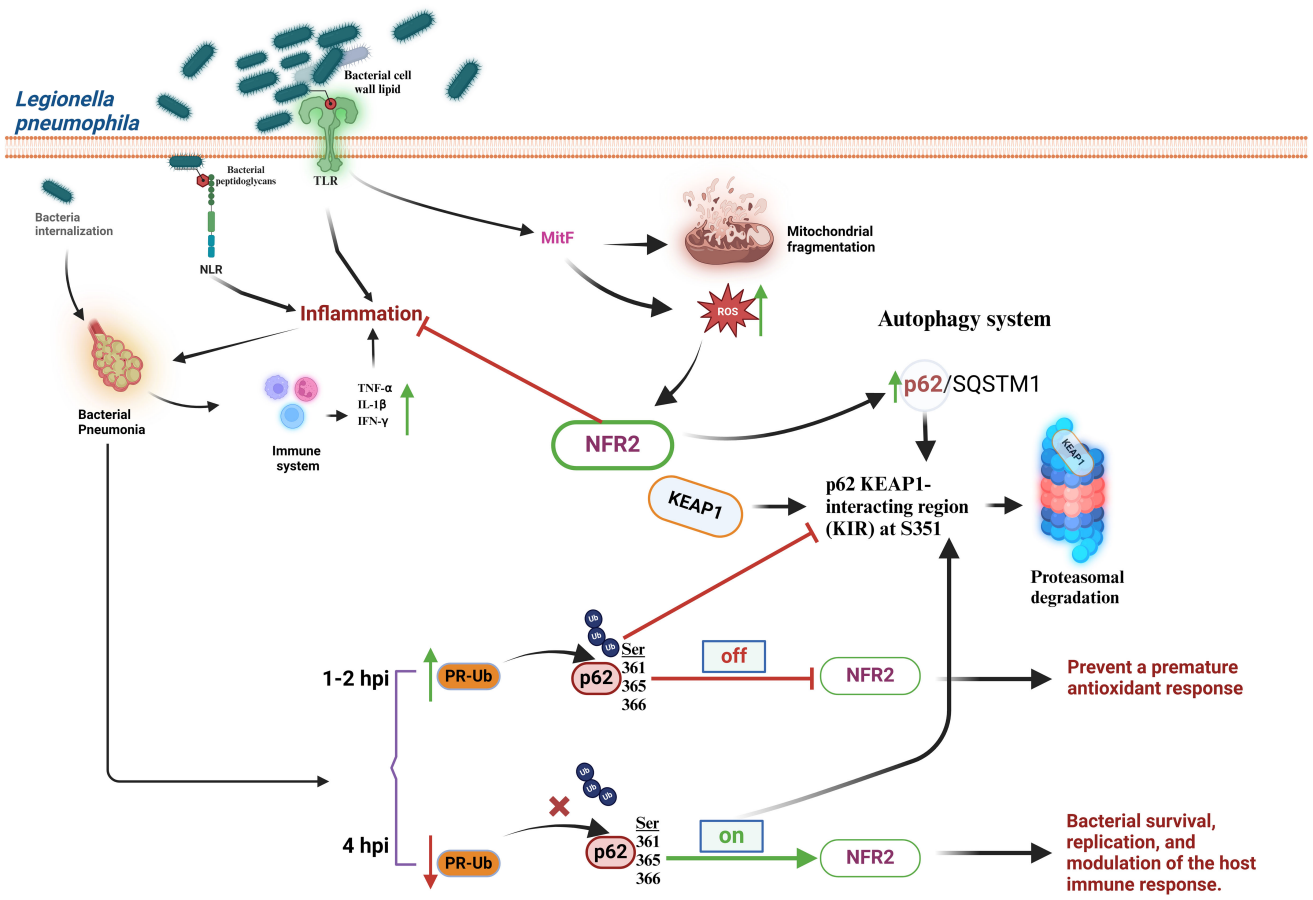
*Legionella pneumophila* manipulates the cellular antioxidant response at initial and later stages of infection. *L. pneumophila* interacts with TLRs or NLRs, triggering the synthesis of the pro-inflammatory cytokines TNF-α, IL-1β, and IFN-γ, increase in ROS production, activation of Nrf2, and mitochondrial fragmentation. Nrf2 may also be linked to an increase in the p62/SQSTM1 autophagic system by promoting the interaction of the Keap1-interacting region of p62 (KIR) with Keap1. At early stages (1-2 h post-infection, hpi) (Off) *L. pneumophila* blocks activation of Nrf2 in a process dependent upon the activity of ubiquitin-linked phosphoribosyl (PR-Ub). At later stages (≥ 4 hpi) (On) a reduction of p62 ubiquitination is observed because the PR-Ub levels decrease. This allows p62 to interact with Keap1 and block Nrf2 degradation. IL-β, interleukin-1beta; IFN-γ, interferon gamma; Keap1, Kelch-like ECH-associated protein 1; KIR, the p62 KEAP1-interacting region; MitF, mitochondrial fragmentation factor; NLRs, Nod-like receptors; Nrf2, nuclear transcription factor erythroid 2-related factor 2; p62/SQSTM1, p62 protein, also called sequestosome 1; PR-Ub, ubiquitin-linked phosphoribosyl; ROS, reactive oxygen species; TLRs, Toll-like receptors; TNF-α, tumor necrosis factor-α.

*S. aureus* is an intracellular bacterium that causes a wide range of infections in humans, ranging from mild skin to severe and life-threatening conditions such as pneumonia, sepsis, and endocarditis. The interaction between *S. aureus* and the host immune system involves various molecular and cellular pathways, including the Keap1/Nrf2/ARE. Although this bacterium may activate the production of ROS that ultimately could lead to Nrf2 activation (**Figure 2-1**), some studies have proposed that *S. aureus* may have developed mechanisms to manipulate the host immune responses for its survival and proliferation, including the modulation of the Nrf2 activity (30). An interesting model to investigate these type of mechanisms is bovine mastitis where a weak immune response is often observed during *S. aureus* infections. In primary bovine mammary epithelial cells (pbMECs) the presence of *S. aureus* was unable to either induce changes in Nrf2 activity or induce the expression of the Nrf2-dependent genes NQO1 and GCLM even when ROS concentration in the infected cells is high (31). The idea that *S. aureus* is able to manipulate the Nrf2 pathway arose from the observation that macrophages (Raw 264.7 cells) expressed HO-1 when stimulated with peptidoglycan (PGN), suggesting the activation of Nrf2 and implicating that the whole bacteria is needed to repress Nrf2 activation as it was observed in pbMECs (32). Activation of the MAPK/p38 signaling pathway in THP-1 macrophages infected with *S. aureus* triggers the production of the proinflammatory cytokines IL-6, IL-1β, and TNF-α (**Figure 2-2**). Remarkably, *S. aureus* took advantage of these cytokines to promote its own intracellular survival (33, 34). An even more surprising strategy is the sequestration of the p62/SQTM1-Keap1/Nrf2 signaling pathway, which normally protects cells from oxidative stress. In this way, *S. aureus* suppresses the macrophage´s antioxidant response and builds up a favorable environment for their replication inside cells (**Figure 2-3**). Consequently, in the initial stage of the infection this bacterium avoids elimination and is able to persists for longer times within host cells. A possible mechanism explaining this behavior was elucidated in pbMECs infected with *S. aureus*. Such study showed that Keap1 was not degraded despite an increase in p62 protein levels (31), which suggests that *S. aureus* can manipulate p62 during an early phase of infection by reducing the phosphorylation levels of p62 at Ser349. It was later demonstrated that p62 phosphorylation affects the Keap1 inactivation machinery, resulting in repression of Nrf2 without necessarily compromising intracellular Keap1 levels. These data were complemented with results obtained from THP-1 macrophages infected with *S. aureus* and subjected to repetitive magnetic stimulation (rMS). In this model, there was a decreased phosphorylation of MAPK/p38 and an increased phosphorylation of p62 at Ser349, which in turn increased its binding affinity to Keap1. The Keap1-p62 complex was degraded by autophagy, allowing the release of Nrf2 that can freely translocate to the nucleus (**Figure 2-4**) (35). Interestingly, NF-κB activity, that is needed to develop a proper immune response and pro-inflammatory cytokine production, is only achieved when Nrf2 was overexpressed in A549 cells, indicating that Nrf2 function is a necessary previous step for a proper NF-κB activation (36). Although a detailed mechanism for this process has not yet been described it is worth mentioning that Keap1 was able to downregulate NF-κB through its interaction with IKKβ. This event ultimately led to IκBα stabilization, suggesting that *S. aureus* manipulates both pathways by preventing Keap1 degradation (**Figure 2-5**).

Roles for the Keap1/Nrf2/ARE pathway in *S. aureus* infections have also been reported in a pneumonia mouse model, where Nrf2 promoted the activation of mitochondrial biogenesis. These events resulted in a limitation of acute lung injury and pro-inflammatory cytokine production that rescued mice from lethal sepsis (37). According to this study, when Nrf2^-/-^ mice were infected with *S. aureus*, a delayed expression of HO-1 and superoxide dismutase 2 (SOD2) was accompanied with an overexpression of TNF-α, IL-1β;, and chemokine ligand 2 (CCL2; from the CC motif subfamily) in the lungs as compared to WT mice. Moreover, in the Nrf2^-/-^ mice lungs the level of IL-10 expression was lower, reinforcing the idea that the absence of Nrf2 promotes a pro-inflammatory environment with more intense infiltration of PMN cells, edema, and loss of alveolar membrane integrity. Of note, in contrast to the mastitis model, the *S. aureus* lung infection is associated with downregulation of p62 levels, leading to the autophagy reduction of damaged mitochondrial proteins that are essential for tissue repair.

It is clear that Nrf2 activation in *S. aureus* infected tissues has a prominent role as a cytoprotective factor and aids to develop a proper immune response. This affirmation has been strengthened by data obtained in studies analyzing the sulforaphane (SFN, an activator of Nrf2) effects in cases of antibiotic resistant to *S. aureus* infections. SFN reduced the internalization and survival of *S. aureus* although both NQO1 and HO-1 expression was not modified (30). These data support the idea that *S. aureus* is able to harness the Nrf2 signaling to extend its survival inside cells.

### Streptococcus pneumoniae

Extracellular pathogen *S. pneumoniae* is a leading cause of community-spread pneumonia. Recently, different studies have pointed out the role of Nrf2 activity during infections caused by this bacterium. In 2020 Maurice et al. showed an increase in the expression of anti-oxidant proteins like Nrf2, in response to mitochondrial-derived oxidative injury in a model of human primary bronchial epithelial cells infected with *S. pneumoniae* (38). In a subsequent study and using a Nrf2^-/-^ mouse model of pneumonia, this bacterium caused epithelial barrier dysfunction accompanied by enhanced neutrophil recruitment to the alveolar space, lung injury, and leukocytosis as well as a high mortality rate in the knock-outs relative to the wild-type animals, suggesting that Nrf2 activation exhibits a protective mechanism during *S. pneumoniae* infections (39). These results were strengthened in several models of pneumonia and keratitis, in which the Nrf2 activators, tert-butylhydroquinone (tBHQ) and resveratrol, down-regulated *S. pneumoniae* inflammation, lung injury, and promoted catalase function that counteracted the H_2_O_2_ secreted by this bacterium (40, 41). Infection of human corneal epithelial cells by *S. pneumoniae* showed that tBHQ down-regulated ROS production, protected cells from cytotoxic effects, and induced Nrf2-dependent autophagy to enhance bacterial clearance. Furthermore, inhibition of IKKβ; activity by free Keap1 contributed to reduce the inflammatory response mediated by NF-κB (**Figure 3A**). Notably, tBHQ also promoted LL-37 antimicrobial peptide expression although no relation to Nrf2 was established (41).

The importance of the genes transcribed under the Nrf2 control in infections caused by *S. pneumoniae* was also observed in macrophages isolated from chronic obstructive pulmonary disease (COPD) patients. These macrophages showed a defect in *S. pneumoniae* phagocytosis that was related to impaired transcriptional response, including the Nrf2 pathway. Phagocytosis capacity was recovered by stimulation of COPD-derived macrophages with the Nrf2 agonists SFN and compound-7 (42). Moreover, when Raw 264.7 macrophages were stimulated with lipoteichoic acid (LTA) from Gram-positive bacterial cell wall, esculetin (a compound from *Dipteryx odorata*) down-regulated nitric oxide synthase (iNOS), nitric oxide (NO) and the NF-κB pathway while promoting Nrf2 activation, which mitigated LTA-induced inflammation by NF-κB activity inhibition (43).

Nrf2 also regulates gene expression of the NADPH-generating enzyme, malic enzyme 1 (ME1) (44). Malic enzyme 1 is essential in fatty acid synthesis, catalyzing the reversible decarboxylation of malate to pyruvate while regenerating NADPH from NADP (45). Reduced ME1 expression in alveolar macrophages (AM) and peripheral blood monocyte-derived macrophages (MDM) from patients with chronic obstructive pulmonary disease (COPD) has been linked to defects in the phagocytosis process of bacteria such as *Haemophilus influenzae* and *Streptococcus pneumoniae* (42, 46). Macrophages exhibited reduced transcriptional responses to opsonized bacteria, including Nrf2-mediated oxidative stress responses, and have defects in efferocytosis, phagocytosis, glycolytic reserve, and metabolic plasticity. The altered responses were reversed by treatment with Nrf2 agonists such as sulforaphane and compound 7, capable of rescuing ME1 expression, thus demonstrating that this enzyme can become a new therapeutic target against these bacterial infections through Keap1-Nrf2 PPI inhibitors (42, 46).

Altogether these data demonstrate that activation of Nrf2 is beneficial to the host during *S. pneumoniae* infection and highlights that activation of the Nrf2 pathway may be a novel strategy to counteract the damage caused by this bacterium.

### Mycobacterium tuberculosis

Infections caused by intracellular pathogen *M. tuberculosis* stand as a prominent global cause of mortality, posing a significant threat to public health. During the course of infection, *M. tuberculosis* induces a disruption in redox balance and compromises the efficacy of protective immunity and emerging evidence suggests that Nrf2 plays a pivotal role in the host response to mycobacterial infections (47, 48). On one hand, Nrf2 activation has been associated with increased antimicrobial activities, promoting the clearance of mycobacteria. This involves the upregulation of autophagy genes, inflammation, and immune modulation. On the other hand, persistent activation of Nrf2 may lead to an immunosuppressive environment, facilitating mycobacterial survival and exacerbating chronic infections (**Figure 3B**) (49). Additionally, RNA-seq and immunoblotting data revealed an elevation in Nrf2 gene expression following THP-1 macrophages infected by *M. tuberculosis* (49). However, longer *M. tuberculosis* exposure caused a decline in Nrf2 protein levels while the expression of Keap1 protein, a negative regulator of Nrf2, remained consistently low. Furthermore, SFN (sulforaphane) induced a pro-inflammatory cytokines reduction, phagocytosis, host cell apoptosis, ROS increase, and autophagy of THP-1 infected with *M. tuberculosis*. Notably, SFN-activated Nrf2 stimulated an enhancement in the THP-1 capacity to eliminate bacteria, possibly through the modulation of protective immunity mediated by Nrf2. It seems that Nrf2 acts like a two-sided weapon against mycobacterial infections (**Figure 3C**). That is, while Nrf2 activity may help clearing *M. tuberculosis* in short term infections, in longer term cases a reduction of Nrf2 levels is observed. Understanding the complex mechanistic behavior of Nrf2 regulation in *M. tuberculosis* infected tissues may guide to novel therapeutic strategies to combat these infections.

### Listeria monocytogenes

*L. monocytogenes* is a motile, rod-shaped, and intracellular facultative anaerobic bacterium causative of the foodborne illness known as listeriosis. *L. monocytogenes* is unique among the pathogenic bacteria because it is able to survive and proliferate at low temperatures (50). This bacterium disrupts the Nrf2 pathway to evade the host immune response through as-yet-unknown mechanism that involves the activation of the immunoglobulin superfamily T-cell membrane protein mucin-3 (Tim-3). Tim-3 has been recognized as an immune checkpoint inhibitor, and its dysregulation has been associated with T-cell tolerance and various immune disorders, including tumors and tolerance to infections (51). However, a precise role of Tim-3 in innate immunity has so far remained unclear. A recent report revealed that Tim-3 hinders the phagocytic activity of macrophages against *L. monocytogenes* by inhibiting the Nrf2 signaling pathway, whose consequence is an increase in bacterial burden. It is likely, as it is observed in some type of cancers, that Tim-3 signaling pathway facilitates Nrf2 degradation through the activation of the tripartite motif (TRIM) family proteins known to induce E3 ligase-dependent but Keap1-independent ubiquitination giving rise to a reduction of Nrf2 nuclear translocation (**Figure 4**) (52, 53). Interestingly, downregulation of two Nrf2-dependent genes CD36 and HO-1 were identified as responsible for *L. monocytogenes* phagocytosis and increased bacterial survival. Restoration of CD36 and HO-1 expression in macrophages reduced bacterial burden and severity of infection (52). These data indicate that downregulation of the Keap1/Nrf2 pathway increases bacterial survival, which suggests that activation of Nrf2 may be a useful strategy to improve *L. monocytogenes* clearance from infected tissues.

## Gram negative bacteria

### Escherichia coli

*E. coli* is a bacterium susceptible to natural and random genetic alteration. Most of the pathogenic *E. coli* strains are extracellular, although some, such as uropathogenic *E. coli*, can become intracellular under certain conditions (54). Hence, it is common to find variants within the same species that are divided into those causing diarrheal and extra intestinal infections. These variants can be facultative or obligate pathogens causing infections that range from moderate to lethal cases (55). Also, normal intestinal microbiota of both humans and animals contains non-pathogenic variants of this bacterium (55). To date, nine pathotypes have been isolated from humans suffering diarrheal and extra intestinal disease (56). However, urinary tract infections (UTIs) caused by uropathogenic *E. coli* (UPEC) are the most common variant worldwide, with an estimate of about 150 million cases per year (57).

Several studies have shown that *E. coli* infections are associated with ROS oxidative stress (58–61). That is why UTI-UPEC are commonly accompanied by an increase in lipid peroxidation and a decrease in the activity of the Nrf2-dependent antioxidant enzymes such as catalase (CAT) and superoxide dismutase (SOD) (62). Furthermore, in a mouse model of acute lung injury (ALI) caused by *E. coli*, it was found an increase in Keap1, low expression of the antioxidant enzymes NQO1and HO-1, and low levels of Nrf2 (63). Reversion of these effects was observed in mice treated with the anti-inflammatory isoquinoline alkaloid sinomenine (SIN) (64). These mice presented a down-regulation of Keap1 expression, an increase in the levels of Nrf2, and high levels of the antioxidant enzymes NQO1 and HO-1. SIN also down-regulated the expression of the pro-inflammatory mediators IL-6, IL-1β, and TNF-α that characterize acute inflammation in *E. coli*-ALI through a mechanism that inhibits the NF-κB nuclear translocation by blocking phosphorylation of IκBα. It seems that SIN significantly ameliorated *E. coli-*induced histopathological changes by reducing lung injury in mice in an Nrf2-dependent manner (63). Nrf2 also induced overexpression of NQO1 in response to LPS during infections caused by *E. coli* in lung tissues and THP-1-derived macrophages (63–66). In this case it was observed a reduction of the NF-κB signaling activity that resulted in lowering the expression of the pro-inflammatory genes IL-6 and IL-1β (63, 65–67). In another study, activation of Nrf2 nuclear translocation after p62 binding to Keap1 in urothelial cells infected with *E. coli* enabled bacterial exocytosis (68). Once in the nucleus, Nrf2 interacted with the promoter region of the RAB27B gene thereby regulating the expression of this protein (68). An important aspect of this work was that increased expression of NQO1, glutamate-cysteine ligase catalytic subunit (GCLC), and HO-1 was required to mitigate the effects caused by ROS. It was also necessary a constant increase in Nrf2 expression to reduce the bacterial load of intracellular *E. coli* in bladder epithelial or urothelial cells (68).

From these results it is evident that modulation of Nrf2 activity in UTI caused by *E. coli* is undoubtedly of paramount importance. Nevertheless, more studies are needed to provide a deep insight of these mechanisms in order to design possible therapeutic strategies that help to control this type of infections.

### Helicobacter pylori

*H. pylori* is generally viewed as an extracellular pathogen, but also exhibit intracellular behavior under certain conditions and is considered a major risk factor in the development of peptic ulcers and gastric cancer. After infection, several virulence factors trigger a transformation process in gastric cells, causing intestinal injury that leads to the appearance of peptic ulcers, atrophic gastritis, intestinal metaplasia, dysplasia, and adenocarcinoma (69). It is estimated that more than 50% of the world’s population is infected by this bacterium, although only about 10% of infected patients develop gastric cancer, suggesting the existence of defense mechanisms by the host against *H. pylori* (70). The inflammatory response initiated by *H. pylori* is closely related to secretion of virulence factors such as cytotoxin-associated gene A (CagA), type IV secretion system (T4SS) (71), vacuolating cytotoxin A (VacA), ureases, adhesins, peptidoglycan outer membrane proteins (i.e., BabA, SabA, OipA, HtrA, DupA, IceA), arginase, γ-glutamyl transpeptidase (GGT), catalase, and SOD (72).

An important toxin secreted by *H. pylori* is VacA, which has the ability to induce formation of vacuoles, induce apoptosis, improve colonization capacity in the stomach, contribute to the pathogenesis of gastric adenocarcinoma, peptic ulcer, and autophagy (73). VacA-activated autophagy is believed to contribute to limiting toxin damage to the host cell (74) and induce oxidative stress, which results in membrane pores formation with subsequent loss of mitochondrial activity (75). The importance of oxidative stress has been widely discussed in the context of gastric cancer (GC) (76), which suggests a highly relevant role of Nrf2. In this scenario, polymorphisms present in the Nrf2 promoter are known to be associated with the development of gastric mucosal inflammation, either independently or associated with *H. pylori* infection (77). By using three different gastric epithelial cell lines (non-cancerous HFE-145, diffuse gastric cancer AGS, and intestinal GC subtype MKN74) Bacon et al. (2022) were able to demonstrate that Nrf2 signaling pathway was differentially regulated depending on the stage of *H. pylori* infection. Moreover, VacA-dependent downregulation of Nrf2 was observed after 24 h of infection, which resulted in an increase in epithelial-to-mesenchymal transition (EMT) an important stage in the development of GC (78). These results suggest that Nrf2 is crucial for maintaining a normal epithelial gastric cell phenotype.

Regulation of Nrf2 during *H. pylori* infection could also be related to an increase in ROS production, which in turn triggers a p62 -Keap1/Nrf2/HO-1 selective autophagy. This process is regulated by the ubiquitination of p62 and by sequential phosphorylation reactions (79). Two important facts are worth to mention on the Keap1/Nrf2/HO-1 signaling axis participation in the autophagy process activated by *H. pylori* in human gastric adenocarcinoma (HGA) that allows oxidative stress-damaged epithelial cells to be eliminated (70): 1) There was a nuclear translocation of Nrf2 upon co-incubation of HGA cells with *H. pylori* for a period of 3 h and 2) Nrf2 induced the expression of HO-1 whose byproduct is carbon monoxide (CO), a molecule considered antioxidant and anti-inflammatory. Therefore, in *H. pylori* infections the Keap1/Nrf2 signaling pathway has been considered as one of the cellular defense mechanisms capable of regulating the autophagy process by modulating the p62 protein. Although the molecular mechanisms in *H. pylori* infections are yet to be fully elucidated, therapeutic approaches targeting Nrf2 seem quite attractive.

### Legionella pneumophila

*L. pneumophila* is an intracellular pathogen considered one of the main causative agents of acquired pneumonia in the European community (80). This bacterium is widely distributed in the environment, causing sporadic outbreaks of opportunistic infections, particularly in young children and the elderly immunocompromised individuals (81). *L. pneumophila* causes two different types of pathologies: 1) Legionnaires’ disease, which includes lung infection symptoms such as cough, difficulty of breathing, fever, and headache and 2) Pontiac fever, which displays flu-like symptoms but does not involve pneumonia. During the infection process, *L. pneumophila* interacts with several immunomodulatory molecules on the cell surface of infected cells, such as TLRs on the plasma membrane and NLRs in the cytoplasm (82, 83). Activation of these receptors induces an inflammatory process due to excessive production of pro-inflammatory cytokines that may contribute to pneumonia symptoms (83). *L. pneumophila* alters the cellular redox metabolism, which in turns activates the Keap1/Nrf2-dependent antioxidant signaling pathway (84). It is believed that MitF (mitochondrial fragmentation factor), an effector injected by the type IV secretion system of *L. pneumophila* to the alveolar macrophages, is one of the causative agents associated with increased ROS production and mitochondrial fragmentation (85, 86). MitF is a protein produced by mitochondrial accumulation of dynamin-1-like protein (DNM1L) a GTPase essential for the regulation of mitochondrial fission and activator of Ran GTPase (Ras-related nuclear protein) and translocation of RNA and proteins through the nuclear pore complex (85). Furthermore, cellular metabolism and autophagy, being closely related processes to redox signaling, link the p62 autophagic system to Nrf2 activation by inhibiting the interaction with Keap1. This mechanism involves phosphorylation of the p62 Keap1-interacting region (KIR) at Ser351, which increases the affinity of p62 to Keap1 preventing the interaction between Keap1 and Nrf2 (84, 87). However, during the early stages of *L. pneumophila* infection (1-2 h post-infection, hpi) to A549 lung cells, ubiquitination of p62 at residues Ser361/365/366, near the p62-KIR, by ubiquitin-linked phosphoribosyl (PR-Ub) prevents its binding to Keap1. Consequently, Keap1 is free to interact with Nrf2, leading to its rapid degradation by the proteasome 26S. This mechanism prevents premature activation of the Nrf2-dependent redox response. In contrast, at 4 hpi, there is a significant decrease in PR-Ub-p62 levels, which promotes Nrf2-dependent transcriptional activation of target genes because phosphorylated p62 at Ser351 binds Keap1 and blocks its interaction with Nrf2 (**Figure 5**) (84, 88).

Interestingly, the interplay between *L. pneumophila* infection and the host cell redox response seems to be a carefully orchestrated battlefield. This bacterium strategically inhibits Nrf2 activation at early stages of infection by manipulating p62 ubiquitination to prevent an antioxidant response. Such a delay may be beneficial for bacterial survival and replication. In contrast, at later stages *L. pneumophila* induces activation of Nrf2, to exploit, perhaps, the antioxidant mechanisms for its own benefit.

### Pseudomonas aeruginosa

*P. aeruginosa* is an extracellular pathogen, and facultative opportunistic aerobic bacterium typically found in soil or water sources that is associated with hospital-acquired pneumonia and chronic lung infections in cystic fibrosis patients (89, 90). The ability of *P. aeruginosa* to cause infections stems from two key factors: 1) Formation of biofilms that resist components of the host immune system and 2) secretion of virulence factors such as exoproteases, phospholipases, hemolysins, and phenazines that facilitate colonization in the pulmonary epithelium (91–93). One of the main virulence factors of *P. aeruginosa*-induced pulmonary disease is the phenazine pyocyanin (1-hydroxy-5-methyl-phenazine, PCN) because of its ability to induce ROS response. ROS production may accelerate bacterial invasion, alter the immune response, and damage lung epithelial cells (94, 95). PCN undergoes reduction by NADPH, allowing it to react with molecular oxygen and generate superoxide radicals and hydrogen peroxide (94, 96).

**Figure 6:**
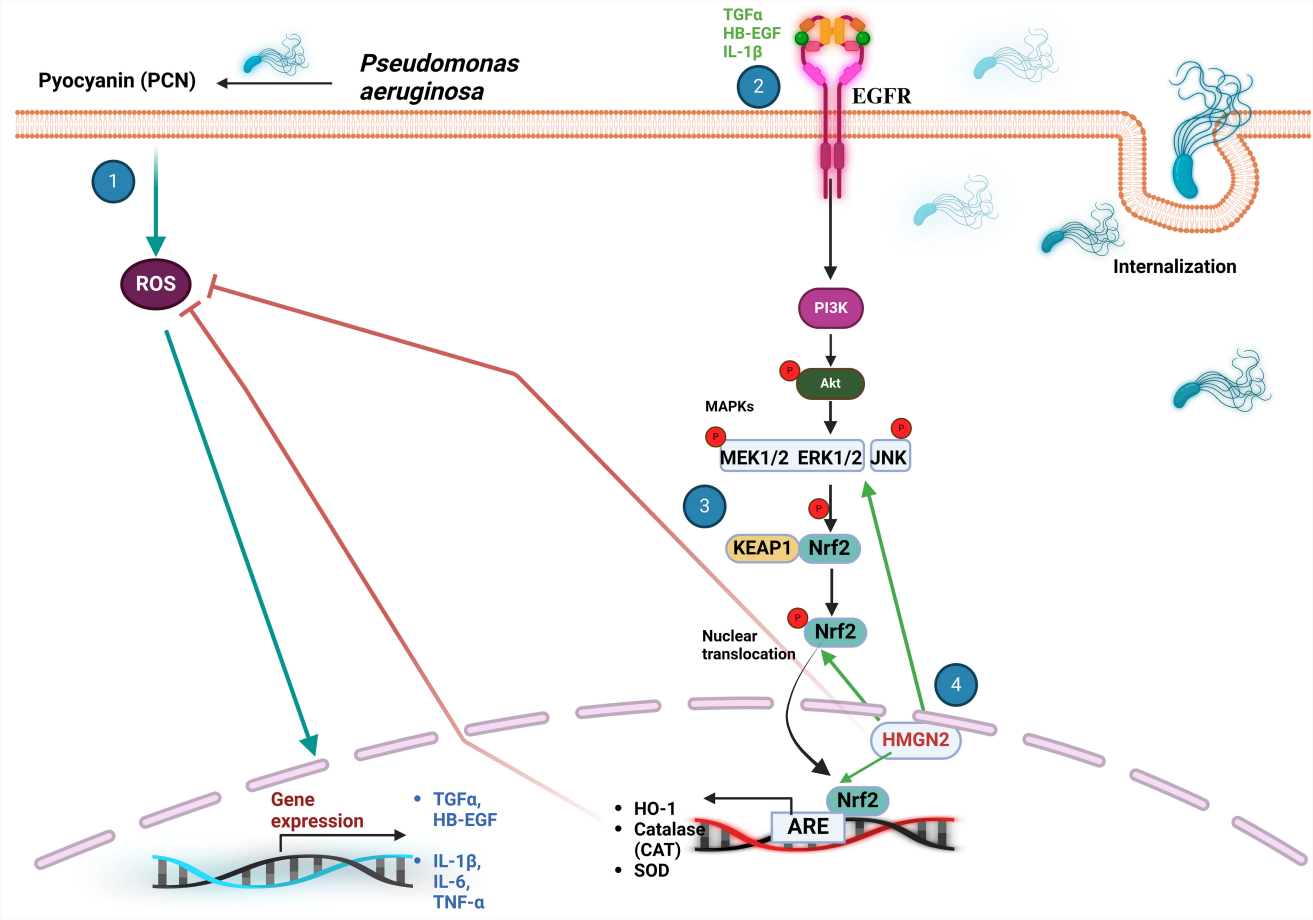
The Keap1/Nrf2 signaling pathway plays a crucial role in the cellular response to *P. aeruginosa* infection. (1) Pyocyanin (PCN) induces an increment in ROS, leading to the expression of TNF-α, IL-6, IL-1β;, TGF-α and HB-EGF that modulate the activity of EGFR. (2) EGFR triggers a downstream signaling cascade that activates PI3K, Akt, the MAPKKK MEK1/2, ERK1/2, and JNK. (3) These protein kinases phosphorylate Nrf2 at Ser215, Ser408, Ser558, Ser577 and Thr559, promoting its nuclear translocation. (4) PCN also activates Nrf2 via HMGN2 that assists ROS scavenging through antioxidant Nrf2-dependent gene expression, actin rearrangement, and activation of ERK1/2 and JNK. ARE, antioxidant response element; Akt, protein kinase B; CAT, catalase; EGFR, epidermal growth factor receptor; HB-EGF, heparin-bound epidermal growth factor; HMGN2, high-mobility group nucleosome-binding protein 2; IL-6 and IL-β, interleukin-6 and interleukin-1beta; JNK, c-Jun N-terminal kinase; Keap1, Kelch-like ECH-associated protein 1; MAPK, mitogen-activated protein kinase; Nrf2, nuclear transcription factor erythroid 2-related factor 2; PCN, pyocyanin; PI3K, phosphatidylinositol-3-kinase; ROS, reactive oxygen species; SOD, superoxide dismutase; TGF-α, tissue growth factor alpha; TNF-α, tumor necrosis factor-α.

Several studies have shown that PCN may be an activator of Nrf2 (97–99). Evidence of Nrf2 accumulation and nuclear translocation was demonstrated to occur in A549 lung cells stimulated with PCN, leading to the expression of the antioxidant Nrf2-dependent genes NQO1 and γ-glutamylcysteine synthetase heavy subunit (γ-GCSH) (98, 100). A proposed mechanism to explain these data suggests that PCN-triggered ROS production causes the expression of Nrf2-dependent genes (HO-1, catalase, and SOD) and TNF-α, IL-6, and IL-1β; that activate the epidermal growth factor receptor (EGFR) either by inducing its expression (TNF-α) (101), by co-regulation of their signaling components (IL-6) (102) or by direct binding (IL-1β;) (103). ROS also induces the expression of ligands such as transforming growth factor alpha (TGF-α) and heparin-bound epidermal growth factor (HB-EGF) that activate this receptor (104). EGFR activates phosphatidylinositol-3-kinase (PI3K), which phosphorylates protein kinase B (Akt) and mitogen-activated protein kinase kinase kinase complex (MAPKKK, MEK1/2) and extracellular signal-regulated MAPK subfamily proteins ERK1/2 (**Figure 6**). These kinases are thought to phosphorylate Nrf2 at specific Ser and Thr residues (Ser215, Ser408, Ser558, Ser577, and Thr559), causing its dissociation from Keap1 and nuclear translocation (101, 105). Redox regulation in lung cells might also involve PCN-induced Nrf2 activation by high mobility group nucleosome-binding protein 2 (HMGN2). These chromatin-associated, non-histone nuclear proteins, play a role in gene transcription, replication, DNA repair, and cell differentiation (98, 106, 107). Nuclear translocation of Nrf2 is among the most important mechanism activated by HMGN2 along with the expression of antioxidant genes, scavenging of ROS, actin rearrangement, and phosphorylation of ERK1/2 and JNK, while regulating *P. aeruginosa* invasion (98).

Despite all these important advances, the exact mechanism of Nrf2 activation by *P. aeruginosa* still remains under investigation. Involvement of canonical and non-canonical Keap1/Nrf2 pathways suggest new possibilities for targeted drug design to combat this and other pathogenic bacteria.

### Salmonella typhimurium

*S. typhimurium*, a rod-shaped and intracellular bacterium, is a major cause of foodborne illness worldwide. It infects both humans and animals, leading to the development of diseases like chronic gastroenteritis (108, 109). During the course of infection, *S. typhimurium* secretes adhesion and virulence factors that are injected to the host intracellular environment by the type III secretion system (T3SS) (110). Notably, the cytotoxic protein SpvB, secreted via T3SS, modulates the inflammatory response by directly targeting the NF-κB signaling pathway. SpvB prevents IKKβ phosphorylation, thereby hindering the subsequent phosphorylation and degradation of IκBα. Ultimately, this represses NF-κB nuclear translocation and pro-inflammatory gene expression, dampening the host’s immune response and promoting bacterial survival and replication (111).

In addition to manipulating inflammation via NF-κB, *S. typhimurium* also induces oxidative stress, which in turn activates the Keap1/Nrf2 pathway. This pathway leads to the expression of genes involved in defense and detoxification, potentially protecting host cells from oxidative damage (112, 113). Recent studies have demonstrated that esculetin (EST), a natural coumarin found in various dietary foods and herbs, can activate Nrf2. This activation effectively inhibits the proliferation and expansion of multidrug-resistant (MDR) *Salmonella Typhimurium*. Notably, EST significantly downregulates the expression of the type 3 secretion system-1 (T3SS-1) in both intestinal epithelial cells (IEC-6) and a murine model (114). These findings suggest that dietary coumarins or a targeted plant-based diet may offer a promising strategy to counteract MDR bacterial-induced enteric diseases through Nrf2 activation.

However, confirming data on targeting the Nrf2 pathway by *S. typhimurium* are limited. For instance, in bone marrow-derived mouse macrophages (BMDM) infected with this bacterium, type I interferon signaling (IFN-I) disrupts Nrf2 responses to oxidative stress, which results in mitochondrial dysfunction and subsequent cell death by necrosis. IFN-I induces a positive regulation of the mitochondrial phosphatase Pgam5 that represses the Nrf2-dependent transcription of antioxidant genes (113). Conversely, mitochondrial damage caused by ROS promotes autophagy of p62, a protein that competes with the Nrf2 binding to Keap1. This further reduces the antioxidant activity of Nrf2 during Salmonella infection (113, 115). In a different study in RAW264.7 mouse macrophages infected with S. typhimurium the SpvB protein disrupted iron metabolism facilitating bacterial survival and replication (116). This work also revealed that the SpvB C-terminal domain targets Nrf2 for degradation via the proteasomal pathway and prevents transcription of ferroportin (FPN), a protein responsible for iron export from macrophages. Consequently, iron accumulates intracellularly, leading to severe serum hypoferremia, cellular injury, and acute inflammatory processes. Additionally, Deng et al. (2022) were able to demonstrate that NLRP6, a member of the nucleotide-oligomerization domain-like receptors (NLR) family, regulating host iron metabolism that perturbs host resistance to bacterial infection in both macrophages and enterocytes through increasing the unique iron exporter ferroportin-mediated iron efflux in a Nrf2-dependent manner during *S. typhimurium* infection (117). In this investigation, it was observed that NLRP6 negatively regulates the activation of Nrf2 by binding to AKT, reducing the dissociation of the Nrf2-Keap1 complex, and the nuclear translocation of Nrf2. Consequently, there is a decrease in the *fpn* gene, which codes for ferroportin (FPN), and a reduction in iron export, as well as an increase in intracellular iron levels that promote bacterial survival. In the absence of NLRP6, AKT phosphorylates p62 at Ser351, mediated by the mammalian target of rapamycin complex 1 (mTOR). This phosphorylation negatively regulates the transcription of Keap1 by promoting the phosphorylation of the transcription factor Forkhead box class O 3a (FOXO3A) while simultaneously activating the nuclear translocation of Nrf2 (117). All these data have allowed us to consider NLRP6 as a new potential therapeutic target to limit bacterial iron acquisition during *S. typhimurium* infections through the regulation of Nrf2.

Overall, the interplay between Salmonella and the Nrf2 pathway is complex. It is evident that more research is needed to fully understand the role of Nrf2 during Salmonella infection. This knowledge could be crucial for developing targeted therapies that modulate the Nrf2 pathway for the treatment of Salmonella-associated diseases.

## Concluding remarks and future perspectives

Harnessing the Nrf2 signaling pathway seems to be a recurrent strategy exploited by a variety of bacteria to facilitate their invasion, survival, and proliferation. In general, the inhibition of the Nrf2-associated antioxidant and anti-inflammatory genes seems to be beneficial in most of the cases discussed here. Nonetheless, the intricate interplay between the microorganism and the host is more complex as observed in infection caused by *S. pneumoniae* and *S. typhimurium*. In these cases, bacterial-mediated activation of the Nrf2 pathway paradoxically benefits the microorganism. A notable finding is the temporal variability in the modulation of the Nrf2 signaling pathway by diverse bacterial species. Both upregulation and downregulation of Nrf2 have been observed at different post-infection time points, highlighting the importance of considering temporal dynamics in future scientific endeavors.

In this context, what seems to be emerging as a common strategy is the fine-tuning timing that bacteria utilize to manipulate various canonical and non-canonical mechanisms to regulate the Nrf2 activity to their own benefit. Understanding not only the particular interactions that each infectious microorganism uses to control the antioxidant response but also the time evolution of such manipulation may delineate the development of novel therapies to tackle these infections.

On the other hand, recent research has demonstrated the participation of Nrf2 as a key regulator of mitochondrial function, which opens new perspectives as therapeutic strategies for the management of bacterial infections. Given the diversity of bacterial virulence factors, many bacteria alter mitochondrial function for their benefit (118). Interestingly, many of these virulence factors specifically target cellular processes that modulate host mitochondrial pathways, such as apoptosis, mitochondrial fusion and fission, energy production, mitochondrial dynamics, mtDNA damage, biogenesis, mitophagy, and the mitochondrial unfolded protein response (118–120). For instance, *Escherichia coli* secretes the T3SS effectors Map and EspF, deregulating mitochondrial morphology and promoting calcium release-mediated apoptosis (121–123). In contrast, *S. flexneri and S. typhimurium* secrete the T3SS effectors IpaD and SipD, inducing mitochondrial depolarization, activation of the non-canonical intrinsic apoptosis pathway, and autophagy-mediated type II programmed cell death, respectively (118, 123). Other bacteria, such as *S. pneumoniae, M. tuberculosis*, and *P. aeruginosa*, cause the release of mitochondrial genetic material (mtDNA), leading to acute lung injury in mouse models (124–126). Additionally, bacteria like *L. monocytogenes* and *S. aureus* suppress mitochondrial ROS, necessary for the elimination of the bacterial agent, and alters mitochondrial biogenesis and the mitophagy process (118, 126).

Antioxidant therapies carried out by activating Nrf2 with molecules such as bardoxolone methyl and rosmarinic acid have been shown to help restore mitochondrial function, leading to increased bacterial clearance and resolution of inflammation through enhanced antioxidant, mitophagy and mitochondrial biogenesis pathways, demonstrating the therapeutic potential of Nrf2 on bacterial infections (127, 128).
